# Bioinformatics analyses for the identification of tumor antigens and immune subtypes of gastric adenocarcinoma

**DOI:** 10.3389/fgene.2022.1068112

**Published:** 2022-12-12

**Authors:** Shuxun Wei, Qiang Sun, Jinshui Chen, Xinxing Li, Zhiqian Hu

**Affiliations:** ^1^ Department of General Surgery, Tongji Hospital, School of Medicine, Tongji University, Shanghai, China; ^2^ Department of General Surgery, The 991st Hospital of Joint Logistic Support Force of People’s Liberation Army, Hubei, China

**Keywords:** mRNA vaccine, immunotype, stomach adenocarcinoma, tumor immune, raw letter analysis

## Abstract

**Background:** Although mRNA vaccines have been effective against multiple cancers, their efficacy against stomach adenocarcinoma (STAD) remains undefined. Immunotyping can indicate the comprehensive immune status in tumors and their immune microenvironment, which is closely associated with therapeutic response and vaccination potential. The aim of this study was to identify potential antigens in STAD for mRNA vaccine development, and further distinguish immune subtypes of STAD to construct an immune landscape for selecting suitable patients for vaccination.

**Methods:** The gene expression and clinicopathological features of patients with gastric cancer were downloaded from The Cancer Genome Atlas (TCGA) and Genotype-Tissue Expression Program (GTEx). 729 samples from GSE66229 and GSE84437 were downloaded through GEO and were used as the validation cohorts. Differential gene expression, genetic alterations and prognosis were analyzed using the R package, cBioPortal program and Kaplan-Meier. The relationship between tumor antigens and immune cells was evaluated and plotted by TIMER. ConsensusClusterPlus was used for consistency matrix construction and data clustering, and graph learning-based dimensional reduction was used to depict immune landscape. WGCNA was used to estimate the relationship between the color modules and immune subtypes.

**Results:** Two overexpressed and mutated tumor antigens associated with poor prognosis and infiltration of antigen presenting cells were identified in STAD, including RAI14 and NREP. The immune subtypes showed distinct molecular, cellular and clinical characteristics. IS1 and IS2 exhibited immune-activated phenotypes and correlated to better survival compared to IS3, while IS3 tumors was immunologically cold. Immunogenic cell death modulators, immune checkpoints, and CA125, and CEA were also differentially expressed among the three immune subtypes. Finally, the immune landscape of STAD showed a high degree of heterogeneity between individual patients.

**Conclusion:** RAI14 and NREP are potential antigens for developing anti-STAD mRNA vaccine, and patients with IS1 and IS3 tumors may be suitable for vaccination.

## Background

Gastric cancer (GC) is a global health problem, with more than one million new cases and an estimated 769,000 deaths in 2020, ranking fifth in global incidence and fourth in mortality. Incidence rates of GC are highest in Eastern Asia and Eastern Europe (Lin et al., 2021; Sung et al., 2021). The preferred method for early GC is endoscopic gastrectomy. Non-early GC is treated with surgery, which should include D2 lymphadenectomy for radical effects (Smyth et al., 2020). Perioperative treatment and adjuvant chemotherapy can provide curative resection and extend the survival time for patients with stage 1B and higher cancers (Sexton et al., 2020). Patients with progressive GC who are treated with combination chemotherapy, median survival is less than 1 year (Fuchs et al., 2019; Shitara et al., 2020; Janjigian et al., 2021). Even with the advances made in the treatment of biologics, anti-angiogenic therapy, and immunotherapy for chemorefractory gastric cancer, the treatment efficacy remains limited and is subject to different tumor biology in various populations (Joshi and Badgwell, 2021).

Immunotherapy has revolutionized oncology treatment, including cytokine therapy, adoptive cell transfer (ACT), cancer vaccine, and immune checkpoint therapy. Cancer immunotherapy acts directly on the tumor microenvironment (TME) and re-engaged the anti-tumor immune response. Patients can achieve significant clinical responses, but only a subset of tumor types can benefit from it (Li et al., 2021). Immune checkpoint inhibitors (ICIs) targeting programmed cell death protein 1 (PD-1) and its ligand 1 (PD-L1) have prolonged median survival in clinical trials with single or multiple drug combinations, but many patients do not suit for the treatment (Shitara et al., 2020; Janjigian et al., 2021; Kwon et al., 2021).

Cancer vaccines is a promising immunotherapy, and sipuleucel-T plays an important role in the treatment of prostate cancer as the first cancer vaccine approved by the American Food and DrugAdministration (FDA). Although there are several types of cancer vaccines, mRNA vaccines have the advantages of providing the complete epitopes and expressing more protein compared with other types of vaccines. With the development of technology in recent years, the difficulties of synthesis, purification, storage and transportation in the development of mRNA vaccine have been overcome. Therefore, using mRNA vaccine to treat gastric cancer is very promising. However, the antigen and potential patients of mRNA vaccine in gastric cancer remain to be studied.

In our analysis, bioinformatics methods was used to screen potential antigens for gastric cancer mRNA vaccines by gene expression level, mutation frequency, association with antigen presenting cells, and prognostic effect. In addition, we constructed 3 subtypes of gastric cancer by combining immune-related genes and consistent clustering method. These subtypes have significant differences in immune cell infiltration, tumor mutation load and prognosis, which can be used as an indicator to select the population suitable for tumor vaccine. Finally, we used the method of pseudo-time analysis and WGCNA to select potential predictors of mRNA vaccine efficacy.

## Materials and methods

### Data collection

The gene expression and clinicopathological features of 336 tumor samples and 36 paracancer samples and 174 normal samples were extracted from The Cancer Genome Atlas (TCGA, https://portal.gdc.cancer.gov/) and Genotype-Tissue Expression Program (GTEx, https://www.gtexportal.org/) through UCSC Xena (http://xena.ucsc.edu/) (Goldman et al., 2020). A total of 729 gastric cancer samples from GSE66229 and GSE84437 were downloaded through GEO DataSets (https://www.ncbi.nlm.nih.gov/gds/) and was used as the validation cohort.

### Data preprocessing

The exclusion criteria of transcriptome samples were as follows: 1) Without survival information; 2) Without age, sex, or the American Joint Committee on Cancer Tumor Node Metastasis (AJCC TNM) stage information; 3) Has received neoadjuvant therapy. Genes with zero Fragments per Kilobase Million (FPKM) in more than half of the samples will be eliminated.

### Identification of tumor antigens

The differentially expressed genes (DEGs) between STAD and normal tissue were identified by the Wilcox test with the criteria of |log2FC| > 1 and *p*-value <0.01. Based on the cBioPortal for Cancer Genomics (cBioPortal, http://www.cbioportal.org), DEGs with significant genetic alterations (*p*-value <0.05) were further selected for survival analysis. Genes with *p*-value < 0.01 in Kaplan-Meier method of overall survival (OS) and recurrence-free survival (RFS) were taken for candidate tumor antigens. The relationship between tumor antigens and immune cells was evaluated and plotted by Tumor IMmune Estimation Resource (Li et al., 2016) TIMER.

### Construction and validation of the immune subtypes

4983 immune-related genes were extracted from published immune gene sets and articles (Bindea et al., 2013; Bhattacharya et al., 2014; Wolf et al., 2014; Newman et al., 2015; Becht et al., 2016; Bhattacharya et al., 2018; Nirmal et al., 2018; Miao et al., 2020). Based on TCGA transcriptome expression profile, a matrix with 3367 immune genes was finally brought into Consensus clustering analysis through R package named ConsensusClusterPlus (Wilkerson and Hayes, 2010). The “1-Pearson correlation” was used to evaluate distance and 500 bootstraps were performed to acquire robust subtypes. The best number of subtypes was decided by the consensus matrix, consensus cumulative distribution function, and the relative change in area under CDF curve. The centroids of TCGA immune subtypes and pearson correlation were used to infer the immune subtypes of two GEO validation cohorts.

### Relationship between immune subtypes and clinicopathological features

To evaluate the relationship between immune subtypes and clinicopathological features, the distribution of stage, lauren classification, CEA and CA125 in different immune subtypes was analysed. The effect of immune subtypes on overall survival was also validated in training cohort and validation cohorts. The number of mutation genes and tumor mutation burden (TMB) were also compared among immune subtypes.

### Immune landscape of STAD

The single sample gene set enrichment analysis (ssGSEA) was used to caclulate the enrichment score of immune cell signatures in each sample (Hanzelmann et al., 2013). Different levels of immune checkpoints (ICPs), immunogenic cell death (ICD) modulators and immune-related signatures among immune subtypes were compared.

The distribution and evolution of STAD samples was evaluated by the Monocle package. When creating the monocle object, lowerDetectionLimit was set to 0.1 and expressionFamily was set to negbinomial. size. Genes with low expression are filtered by detectGenes function. Then the DDRTree method was used for dimension reduction.

### Identification of potential biomarkers for mRNA vaccine

In order to select candidate genes that can potentially predict the effect of mRNA vaccine, the weighted correlation network analysis (WGCNA) (Langfelder and Horvath, 2008) was used to estimate the relationship between the color modules and immune subtypes. The analysis was apply on the 3367 immune-related genes. A gene correlation matrix with a optimal soft thresholding of power 4 was used to derive the adjacency matrix. Modules were obtained with the following criterion: A minimum module size of 30 and a minimum height for merging modules of 0.2.

## Results

### Identification of tumor antigens of STAD

**FIGURE 1:**
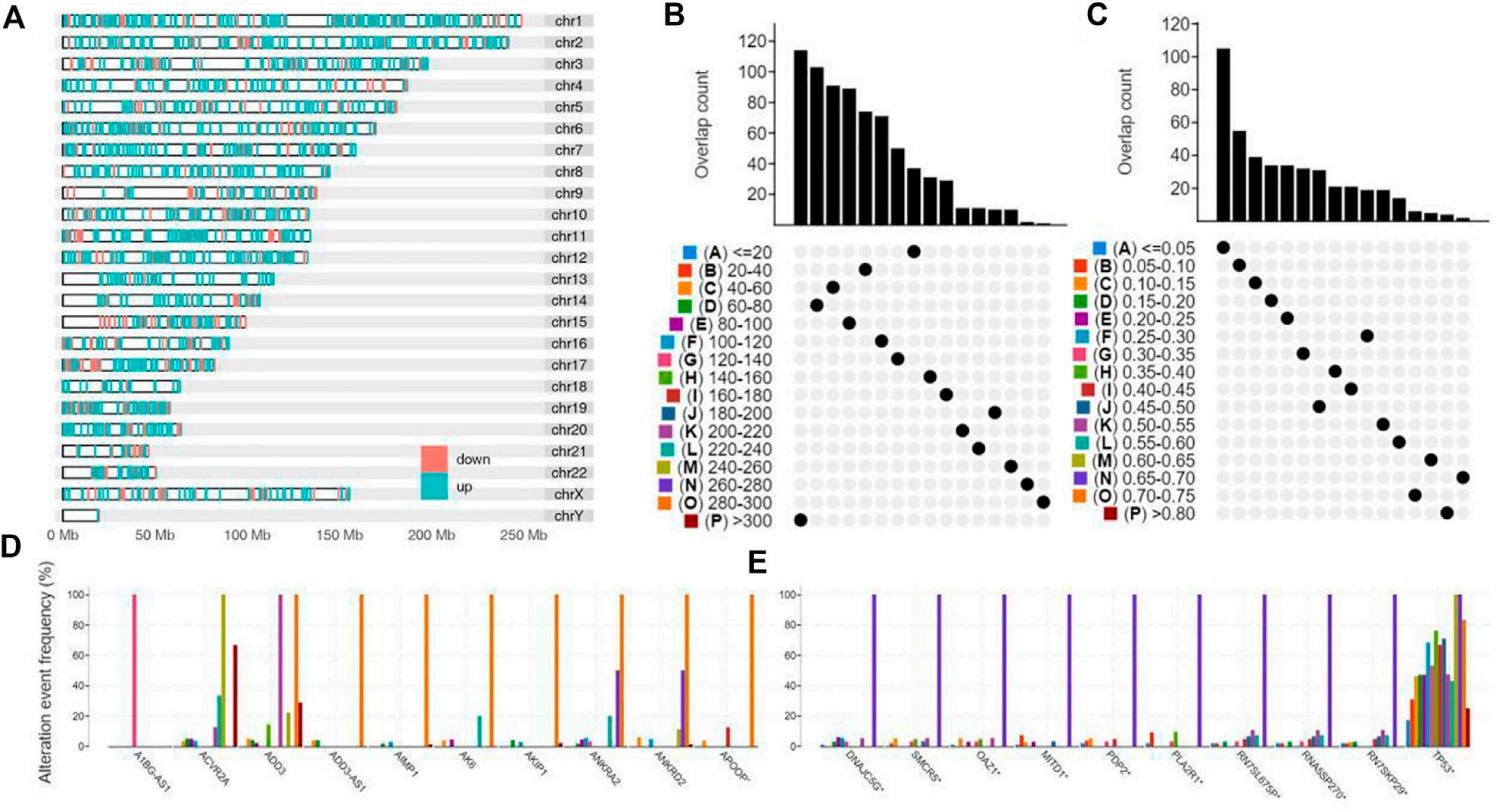
Identification of potential tumor antigens of STAD. **(A)** Identification of potential tumor-associated antigens of STAD. Chromosomal distribution of up- and down-regulated genes in STAD as indicated. **(B–E)** Identification of potential tumor-specific antigens of STAD. Samples overlapping in **(B)** altered genome fraction and **(C)** mutation count groups. Genes with highest frequency in **(D)** altered genome fraction and **(E)** mutation count groups.

2286 upregulated protein-coding differentially expressed genes between STAD and normal tissue were selected for potential antigens of STAD and distributed on different chromosomes (Figure 1A). 3056 genes with a high mutation frequency of altered genome fraction and mutation counts were screened for further analysis (Figures 1B,C). As shown in Figures 1D,E, TP53 gene and RN7SKP29 gene were the most frequently mutated genes. Genes with highly mutation count included ADD3, ACVR2A, and ANKRA2. Synthesizing the above result, 302 intersection genes of upregulated DEGs and highly mutated tumor specific genes were extracted.

**FIGURE 2:**
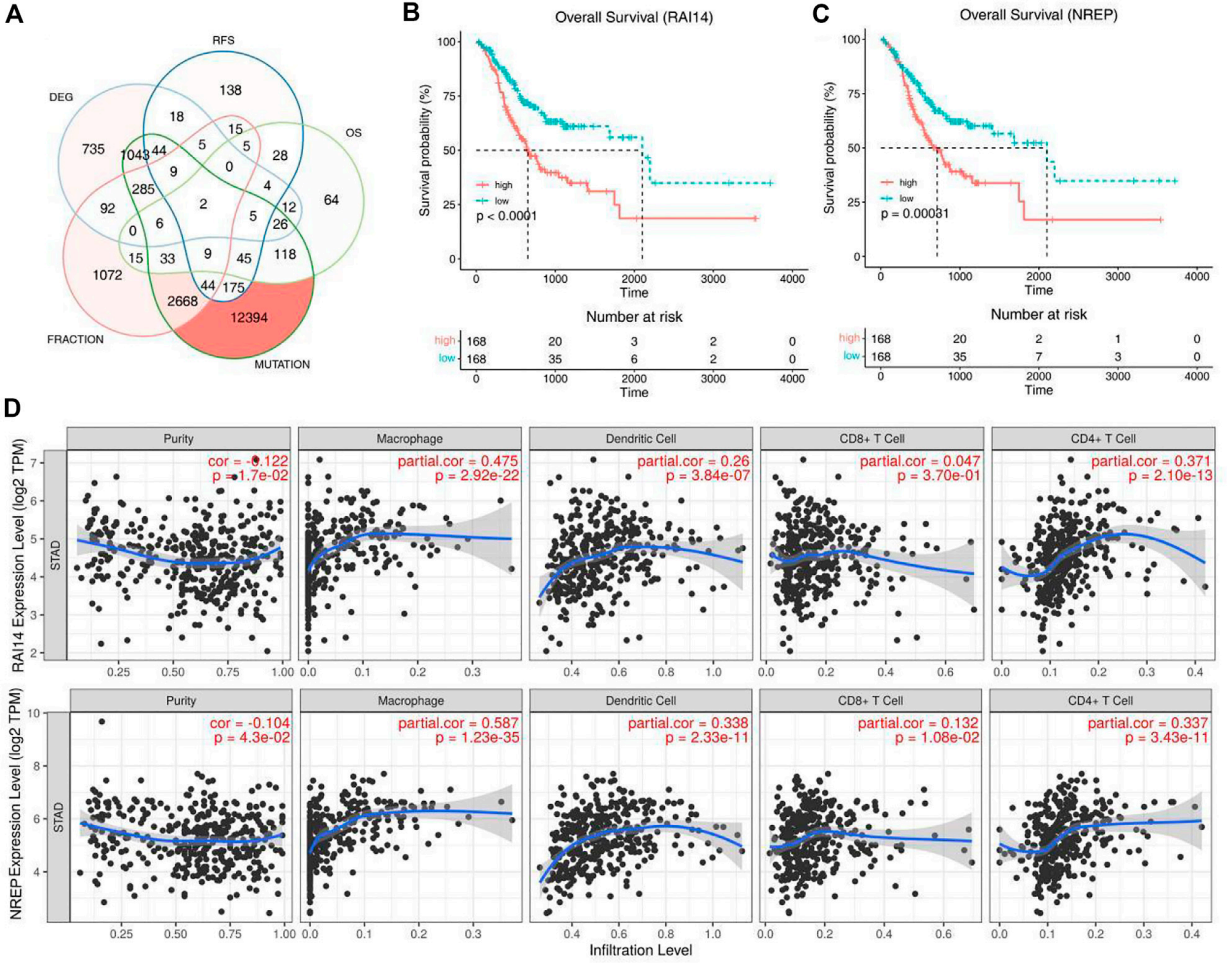
Identification of tumor antigens associated with STAD prognosis. **(A)** Potential tumor antigens with high expression and mutation in STAD, and significant association with OS and RFS (total 2 candidates). **(B,C)** Kaplan-Meier curves showing OS of STAD patients stratified on the basis of **(B)** RAI14, **(C)** NREP expression levels. **(D)** Identification of tumor antigens associated with APCs. Correlation between the expression levels of RAI14, NREP and infiltration of macrophages, dendritic cells, CD8+T cells and CD4+T cells in STAD tumors.

Kaplan-Meier analysis was used to select prognosis-related tumor antigens and 2 genes showed significant correlation with the OS and RFS (Figure 2A). Both of these genes named RAI14 and NREP are oncogenes (Figures 2B,C and Supplementary Figure S1A,B). All of them were positively correlated with macrophages, dendritic cells and CD4^+^ T cells while the expression of NREP was associated with CD8^+^ T cells (Figure 2D). Thus, these results suggest that the 2 prognosis-related potential tumor antigens may be recognized and processed by antigen presenting cells (APCs) and trigger immuneresponse, and were suit to develop mRNA vaccine for STAD.

### Construction and validation of immune subtypes

**FIGURE 3:**
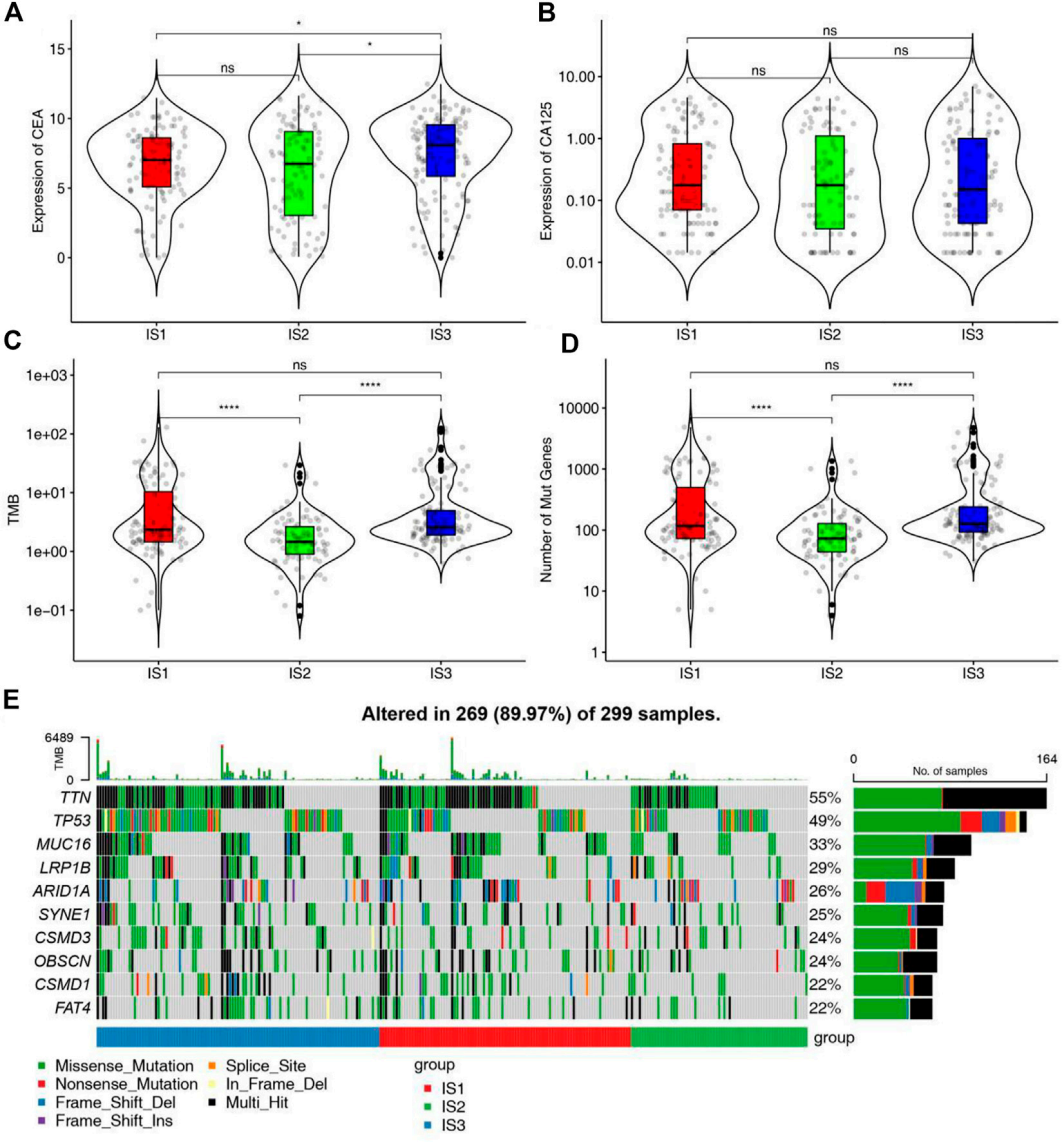
Association between mutation status and different immune subtypes. **(A,B)** Association between immune subtypes and **(A)** CEA and **(B)** CA125 in STAD IS1-IS3. **(C,D)** Association between immune subtypes and **(C)** TMB and **(D)** mutation number in STAD IS1-IS3. **(E)** Ten highly mutated genes in STAD immune subtypes. *p < 0.05 and ****p < 0.0001.

The expression matrix of STAD in TCGA and corresponding 3367 immune genes extracted from published immune gene sets and articles were used as training cohort to construct the immune subtypes. K value was tested from 2 to 10 and consensus clustering was based on 1,000 resampled datasets through the ConsensusClusterPlus package (Supplementary Figures S1A–C, S2G). We found that there was no clear defining feature when we chose k = 4. In the end, we chose k = 3 for the reason that the clustering result could be explained in the context of the biological meaning of STAD. Clustering results were named as IS1-IS3, where IS3 was associated with better prognosis and IS2 predicts a poor prognosis through Kaplan-Meier analysis (Supplementary Figures S1F, S2C). The survival outcome was consistent with the distribution of pathological type and AJCC stage in different clusters. Patients with advanced tumors (stage III/IV) or diffuse pathologic subtype accounted for a high proportion in the IS2 subtype and patients with early stage (stage I/II) or intestinal pathologic subtype accounted for a high proportion in the IS3 subtype (Supplementary Figures S1D,E). Higher levels of CEA and CA125 can be used for diagnosis, prognosis and recurrence of gastric cancer, but the sensitivity and accuracy of both still need to be improved. The expression of CEA and CA125 was no significant difference in different immune subgroups, indicating that immune subtype is superior to the two tumor biomakers in predicting prognosis of STAD patients (Figures 3A,B).

To verify the stability of subgroups, the centroid method was used to predict the immune subtypes of GSE66229 and GSE84437. More than 70% of the samples can be matched to the corresponding immune subtypes. Due to different data sources and gene numbers, some samples did not match the corresponding immune subtypes. Compared with the survival plot, the difference in overall survival among immune subtypes was more obvious in the two validation cohorts (Supplementary Figures S1, S2D). The distribution trend of AJCC stages and pathological types in the validation cohorts was similar to that in the training cohort (Supplementary Figures S1G,H). The above results indicate that Immune subtypes were closely related to clinicopathological features, affecting the overall survival of patients, and has good applicability and extensibility.

### Mutation status in different immune subtypes

Since the mutation status is related to the degree of immune infiltration in tumor, the mutation status of each immune subtypes was analyzed and waterfall plot was shown in Figure 3E. Samples of genes with high mutation frequency had the higher proportion in IS1 and IS3. The number of mutant genes and tumor mutation burden in IS1 and IS3 were higher than those in IS2 (Figures 3C,D). Therefore, IS1 and IS3 may accept higher effectiveness of mRNA vaccine compared to IS2.

### Distinction of immunological cold and hot tumor

**FIGURE 4:**
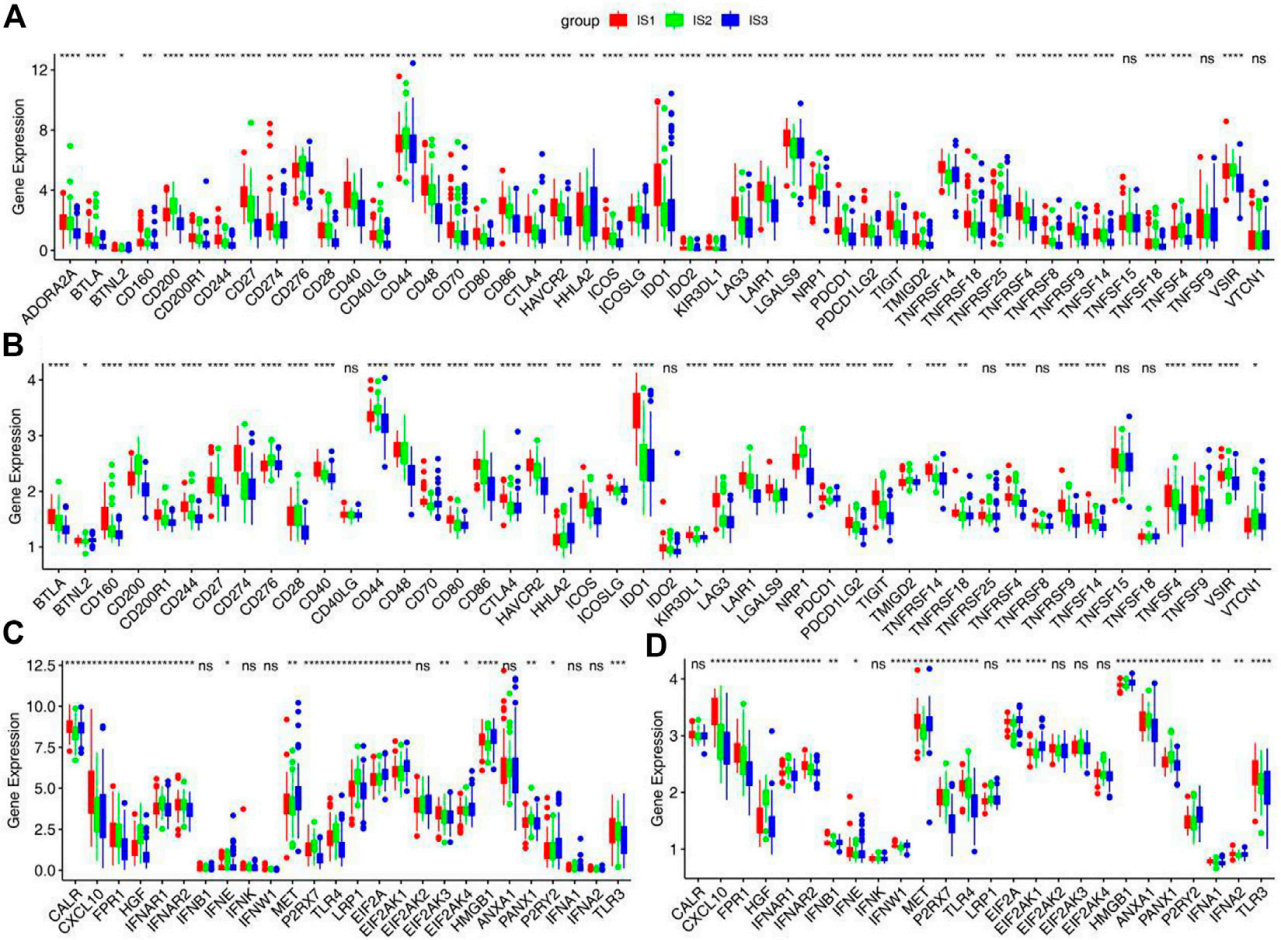
Association between immune subtypes and ICPs and ICD modulators. **(A,B)** Differential expression of ICPs genes among the STAD immune subtypes in **(A)** training cohorts and **(B)** GSE66229 cohorts. **(C,D)** Differential expression of ICD modulator genes among the STAD immune subtypes in **(C)** training cohorts and **(D)** GSE66229 cohorts. *p < 0.05, **p < 0.01, ***p < 0.001, and ****p < 0.0001.

The effectiveness of immune checkpoints (ICPs) depends on the tumor immunity state. The expression levels of ICPs and immunogenic cell death (ICD) modulators in different immune subtypes were calculated. More than 85% ICPs were different with significant statistical difference in different immune subtypes. Most ICPs, such as CD200, CD27, CD40, and CD48, have the same trend, with high expression in IS1 and IS2 and low expression in IS3 (Figures 4A,B and Supplementary Figure S2E). The result shows that immune checkpoint inhibitors may be useless in patients of the IS3. Similarly, more than 75% ICD modulators showed statistically significant differences among immune subtypes. Most ICD modulators have low expression in IS3, but relatively high expression in IS1 and IS2. Similar results can be obtained in two validation sets (Figures 4C,D and Supplementary Figure S2F). These results further suggests that the patients of IS1 and IS2 may have better effectiveness of ICPs and ICD modulators.

**FIGURE 5:**
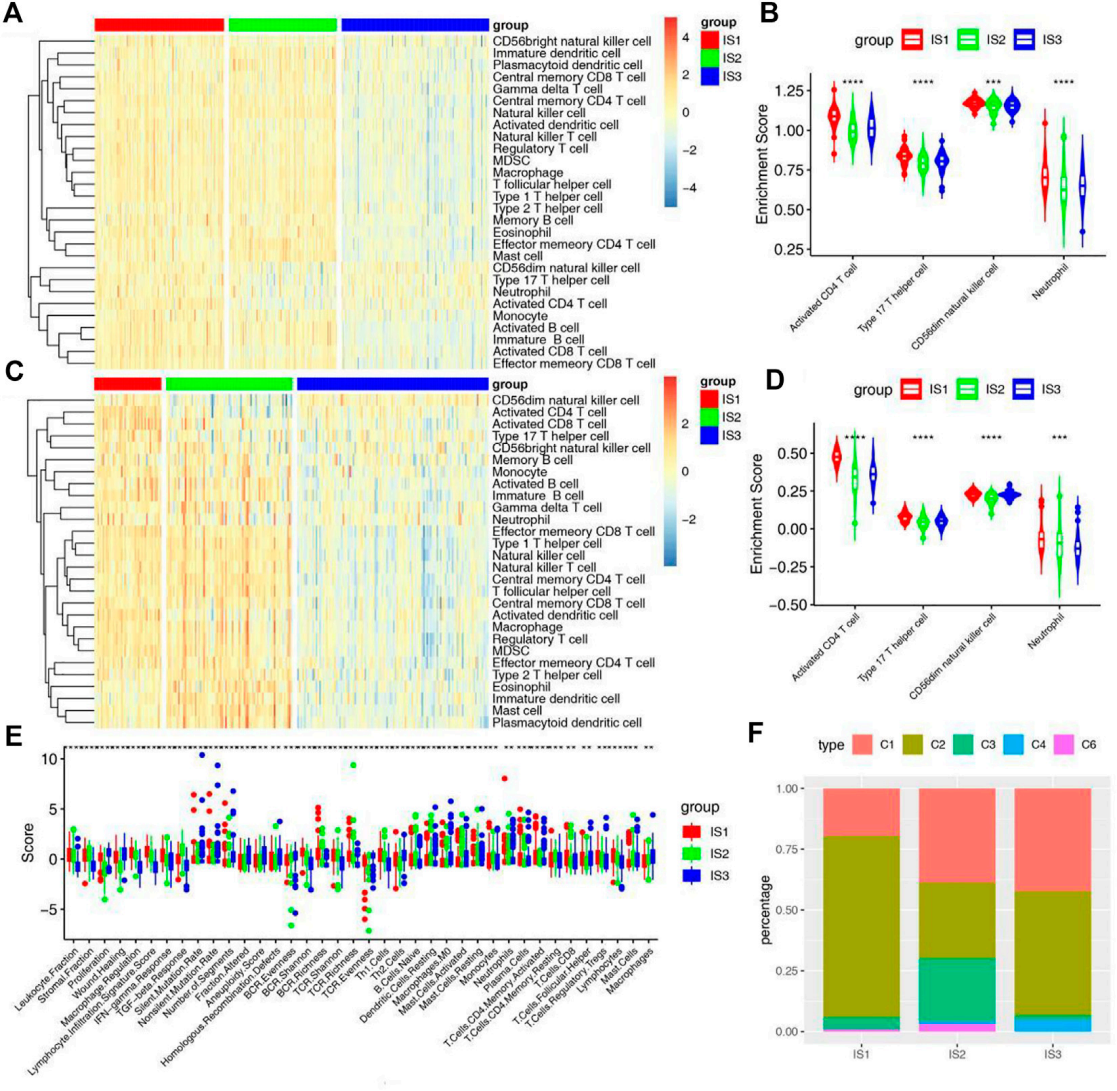
Cellular and molecular characteristics of immune subtypes. **(A)** Differential enrichment scores of 28 immune cell signatures among STAD immune subtypes in training cohorts. **(B)** Differential enrichment scores of 4 prognostically relevant immune cell signatures in training cohorts. **(C)** Differential enrichment scores of 28 immune cell signatures among STAD immune subtypes in GSE66229 cohorts. **(D)** Differential enrichment scores of 4 prognostically relevant immune cell signatures in GSE66229 cohorts. **(E)** Differential enrichment scores of 56 immune signatures among STAD immune subtypes and 38 immune signatures with FDR <0.01. **(F)** Overlap of STAD immune subtypes with 6 pancancer immune subtypes.

To further identify cold and hot tumors, the enrichment of 28 immune cells in samples were analyzed. The heat map shows that almost all immune cells are enriched in the IS1. In the IS2 subtype, most immune cells were significantly enriched except CD56dim Natural killer cell, Type 17 T Helper cell, Neutrophil and Activated CD4 T cell. However, the enrichment of immune cells in IS3 was just opposite to that in IS2. These results were further verified in two validation cohorts (Figures 5A–D and Supplementary Figures S3A,B). Therefore, the IS1 and IS2 are immune “hot” tumors, while IS3 is immune “cold” tumors. To further verify this result, we compared the relationship between these 3 immune subtypes and the 6 immune subtypes previously identified by pan-cancer (Figure 5F) (Thorsson et al., 2018). The results showed that the IS1 mainly included C1, C2and C3, IS2 mainly included C1, C2 and C3, and IS3 mainly contained C1, C2 and C4. Interestingly, C3 is characterized by an inflammatory and C6 is characterized by lymphocyte depleted. These results not only suggest that our classification are consistent with pancancer classification, but also show the reliability of cold and hot classification.

In addition, we further verified the relationship between another 56 molecular signatures and immune subtypes, and identified 38 significantly associated immune-related signatures with FDR <0.01 as the threshold (Figure 5E). The results showed that IS1 had the highest scores for lymphocyte infiltration, IFN-γ and CD8 T cell, IS2 had the highest scores for TGF-β response, monocytes, naive B cell and CD4 memory T cell, and IS3 had the highest scores for wound healing and macrophages. It is suggested that although IS3 is not effective for ICD modulators and ICPs, but it may have a good effect for mRNA vaccines, which brings a therapeutic dawn for patients with immune “cold” tumors.

### Immune landscape of STAD

**FIGURE 6:**
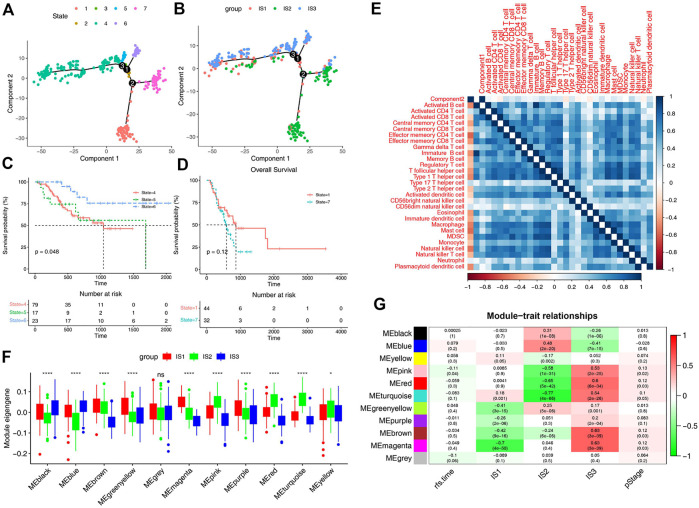
Immune landscape of STAD. **(A)** Immune landscape of STAD. Each point represents a patient and the immune subtypes are color-coded. The horizontal axis represents the first principal component and the vertical axis represents the second principal component. **(B)** Immune landscape of the subsets of STAD immune subtypes. **(C)** Immune landscape of samples from three branches of IS3 and their prognostic status. **(D)** Immune landscape of samples from two branches of IS2 and their prognostic status. Differential enrichment scores of 28 immune cell signatures in the above subsets. **(E)** Heat map of two principal components with 28 immune cell signatures. **(F)** Differential distribution of feature vectors of each module in STAD subtypes. **(G)** Module-trait relationships of STAD immune subtypes.

In order to explore the relationship between immune subtypes and the degree of immune cell infiltration, monocle was used to conduct clustering and pseudotime analysis based on TCGA data. There was a correlation between the two main components and the degree of immune cell infiltration (Figure 6E). It also can be found that IS1 is distributed diffusely, and IS2 is mainly divided into branches 1 and 7, while IS3 subtype is differentiated in the opposite direction to IS2 and mainly distributed in branches 4, 5, and 6 (Figures 6A,B). Survival analysis of these branches showed that there was no significant difference in survival between the two branches of IS2, but the trend of overall survival in branch 1 was better than that in branch 7 (Figure 6D). In the IS3, significant statistical difference was found in the survival analysis of the three branches, and the overall survival in branch 6 was better (Figure 6C). These results suggest that the immune landscape based on immune subtypes is related to the degree of immune cell infiltration and affects prognosis, which is beneficial to the selection of personalized mRNA vaccine.

### Identification of potential biomarkers for mRNA vaccine

**FIGURE 7:**
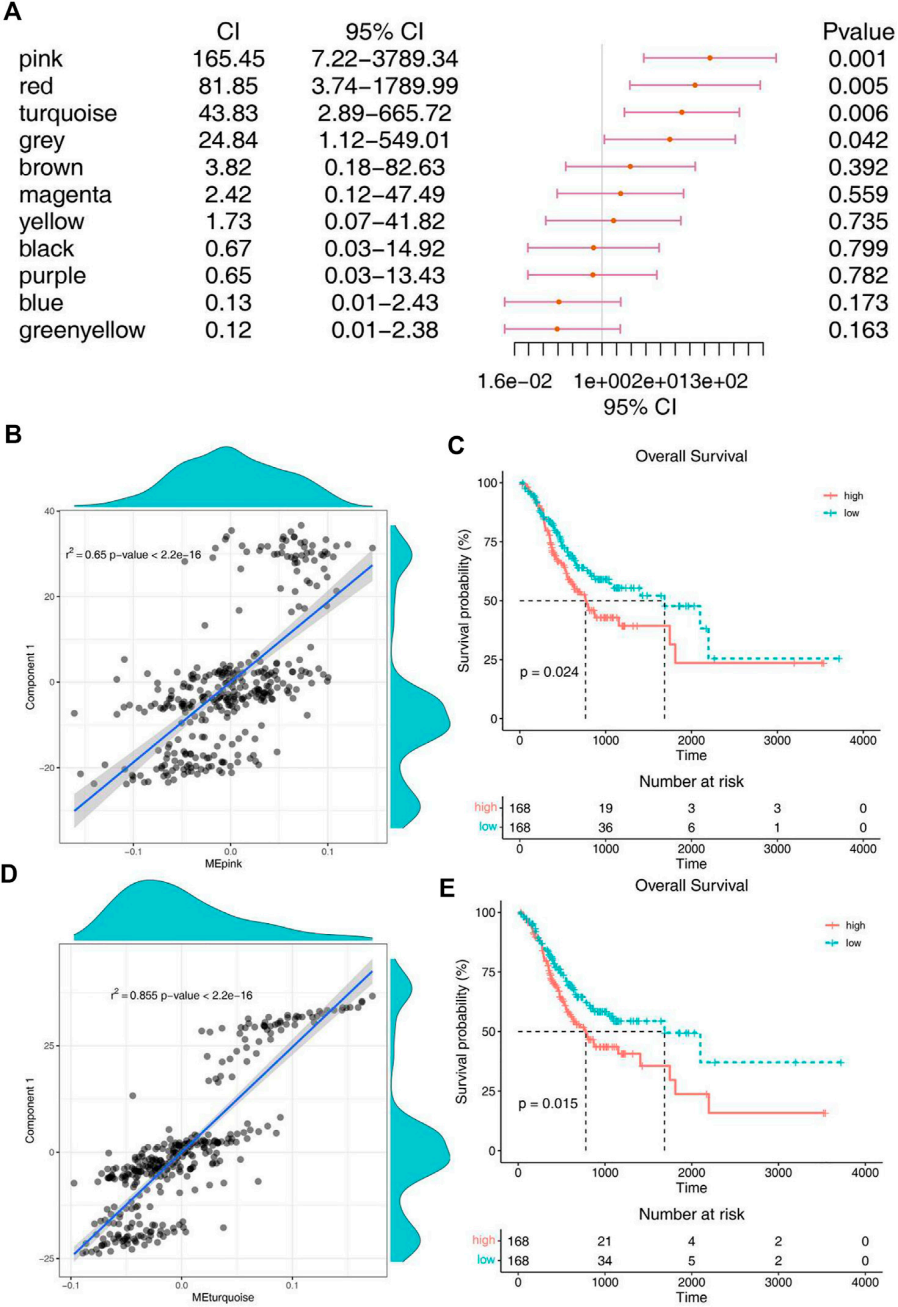
Identification of immune hub genes of STAD. **(A)** Forest maps of single factor survival analysis of 11 modules of STAD. **(B)** Correlation between pink module feature vector and principal component 1 in immune landscape. **(C)** Differential prognosis in pink module with high and low mean. **(D)** Correlation between turquoise module feature vector and principal component 1 in immune landscape. **(E)** Differential prognosis in turquoise module with high and low mean.

For the sake of finding biomakers of mRNA vaccines, WGCNA was used to look for modules associated with immune subtypes. The matrix of 3367 immune genes was analyzed and divided into 11 modules by setting the soft threshold of 4 and the minimum 30 genes for each module (Supplementary Figures S3C–E). Different immune subtypes in different modules have different eigengenes (Figure 6F). We can find that IS1 is highly correlated with magenta module, IS2 is highly correlated with pink, red and turquoise modules, and IS3 is highly correlated with pink, red, turquoise, brown and magenta moduels according to correlation analysis (Figure 6G). Univariate cox regression analysis showed that pink, red and turquoise modules were correlated with prognosis (Figure 7A), while Kaplan-Meier analysis suggest that only pink and turquoise modules were associated with overall survival of STAD (Figures 7C,E and Supplementary Figure S3G). Pink and turquoise modules are highly correlated with principal component 1 of monocle analysis (Figures 7B,D and Supplementary Figure S3F). Combined with the aforementioned results, pink and turquoise modules may be related to the effect of mRNA vaccine, and 14 genes named COL3A1, COL8A1, COL6A3, PDGFRB, MAP1B, KANK2, MAP1A, AOC3, FERMT2, SPARCL1, JAM3, FHL1, AKT3, and ANK2 that has the highest correlation with the two modules may be the potential biomakers of mRNA vaccine.

## Discussion

The first step to explore the application of mRNA vaccines in gastric cancer is to find suitable antigen and suitable population for vaccination. mRNA vaccines work in the same way as traditional vaccines through improving the recognition of antigen presenting cells and further activating the immune response. Based on this principle, we use bioinformatics methods to infer appropriate antigens, which can lay a foundation for our next step work.

You et al. (2022) pioneered the study on immune subtype of mRNA vaccination for STAD and identified ADAMTS18, COL10A1, PPEF1, and STRA6 as potential mRNA vaccine candidates. In this study, we screened samples from TCGA and GTEx databases and performed dual validation in GSE66229 and GSE4437 cohorts to identify a series of targeted antigens. RAI14 and NREP were innovatively found to be promising mRNA vaccine candidates. These two antigens were not only associated with OS and RFS, but also with a variety of APC, and their upregulation was positively correlated with macrophage and DC infiltration. Since mRNA vaccine encoded antigens need to be presented by macrophages and DC, our results suggested that these two mRNA vaccine candidates may play a key role in the tumor biological behavior of STAD. Although these candidate genes must undergo subsequent functional validation, their potential for mRNA vaccine development has been supported by previous reports. For example, several literatures have reported that RAI14 is highly expressed in gastric cancer tissues, and its expression level is correlated with the prognosis of gastric cancer patients. High expression of RAI14 can be used as an independent predictor of poor prognosis in STAD patients (He et al., 2018; Xiao et al., 2020). RAI14 promotes cell growth and invasion, and is regulated by circNFATC3/Mir-23b-3p axis in STAD. RAI14 also plays an important role in the recruitment and regulation of infiltrating immune cells (Yan et al., 2021). Chen et al. (2018) found that RAI14 knockdown could inhibit the proliferation, migration and invasion of gastric cancer cells and promote cell apoptosis by down-regulating the Akt pathway. NREP is highly expressed in embryo and mouse brain, and can participate in cell proliferation, migration, differentiation and other biological functions (Pan et al., 2002; Yao et al., 2015). NREP plays an important role in the progression of malignant tumors and may be involved in the activation of cancer-related fibroblasts and epithelial-mesenchymal transition (EMT), both of which are mediated by transforming growth factor-β1 (Liu et al., 2021). In addition, Liu et al. (2021) reported that the expression of NREP was positively correlated with the abundance of M2 macrophages, which are potent immunosuppressors, suggesting that NREP is overexpressed in GC and affects the prognosis.

In view of tumor heterogeneity, the results of clinical trials of mRNA vaccines have shown benefits for only a small proportion of cancer patients. mRNA vaccines need to be “tailored” to the mutated antigen of patient’s tumor tissue. We developed three immune subtypes of STAD based on immune gene expression matrix and immune genome to select the appropriate population for vaccination. These three immune subtypes showed different molecular, cellular and clinical characteristics. In the training cohort, patients of IS1 showed a better prognosis than the other subtypes, while patients of IS2 showed a worse prognosis, which was generally consistent with the distribution of AJCC stages and pathological types, suggesting that these immune subtypes can be used to predict the prognosis of STAD. There was no significant difference in the expression of serum tumor markers CEA and CA125 in different immune subtypes, indicating that immune subtypes have superior predictive accuracy than these two tumor markers. The mutation status of immune subtype is one of the key factors for the effectiveness of mRNA vaccine, which is related to the degree of immune invasion of tumor. We investigated the molecular and cellular characteristics of the three immune subtypes, and the results showed that the number of mutant genes and tumor mutation burden of IS1 and IS3 were higher than those of IS2, suggesting that patients of IS1 and IS3 may be more responsive to mRNA vaccines.

Most ICPs were highly expressed in IS1 and IS2 and low expressed in IS3, while most ICD modulators were lower expressed in IS3 but relatively higher expressed in IS1 and IS2. These results further suggested the patients of IS1 and IS2 may have better effectiveness of ICPs and ICD modulators. To further characterize the differences in immune status among different subtypes, we studied the enrichment of 28 types of immune cells. Immune cells were most extensively enriched in IS1. In the IS2 subtype, most immune cells were significantly enriched except CD56dim Natural killer cell, Type 17 T Helper cell, Neutrophil and Activated CD4 T cell. However, the enrichment of immune cells in IS3 was just opposite to that in IS2. Therefore, the IS1 and IS2 are immune “hot” tumors, while IS3 is immune “cold” tumors. The correlation analysis of 56 molecular features with immune subtypes also confirmed above result. It is suggested that although IS3 is not effective for ICD modulators and ICPs, but it may have a good therapeutic effect for mRNA vaccines, which brings a therapeutic dawn for patients with immune “cold” tumors.

The complex immune landscape of STAD suggests considerable heterogeneity among individual patients as well as among the same immune subtypes, and by narrowing down immune components, therapies based on personalized mRNA vaccines can be developed. The monocle was used to conduct clustering and pseudotime analysis based on TCGA data. There was a correlation between the two main components and the degree of immune cell infiltration. IS1 was distributed diffusely, and IS2 was mainly divided into branches 1 and 7, while IS3 subtype was differentiated in the opposite direction to IS2 and mainly distributed in branches 4, 5 and 6. There was no significant difference in survival between the two branches of IS2, but the trend of overall survival in branch 1 was better than that in branch 7. In the IS3, significant statistical difference was found in the survival analysis of the three branches, and the overall survival in branch 6 was better. The immune landscape based on immune subtypes is related to the degree of immune cell infiltration and affects the prognosis, which facilitates the selection of personalized mRNA vaccines. Kaplan-Meier analysis suggest that only pink and turquoise modules were associated with overall survival of STAD. Pink and turquoise modules are highly correlated with principal component 1 of monocle analysis, suggesting that patients with high levels of expression of these genes may have a better response to mRNA vaccines. At last, 14 genes named COL3A1, COL8A1, COL6A3, PDGFRB, MAP1B, KANK2, MAP1A, AOC3, FERMT2, SPARCL1, JAM3, FHL1, AKT3, and ANK2 that has the highest correlation with the two modules may be the potential biomakers of mRNA vaccine.

## Conclusion

RAI14 and NREP are potential STAD antigens for development of mRNA vaccines. Patients of IS1 and IS3 may be better candidates for vaccination. mRNA vaccines may also be available for patients with immunologically “cold” tumors. Our results provide a new rationale for developing anti-STAD mRNA vaccines, predicting patient outcomes, and selecting patients for vaccination.

## Data Availability

The datasets presented in this study can be found in online repositories. The names of the repository/repositories and accession number(s) can be found in the article/Supplementary Material.
