# Supplementary figures and images for: Bioinformatics analyses for the identification of tumor antigens and immune subtypes of gastric adenocarcinoma
Source: Front Genet. 2022 Dec 12;13:1068112. doi: 10.3389/fgene.2022.1068112 (PMC9791036; doi:10.3389/fgene.2022.1068112)

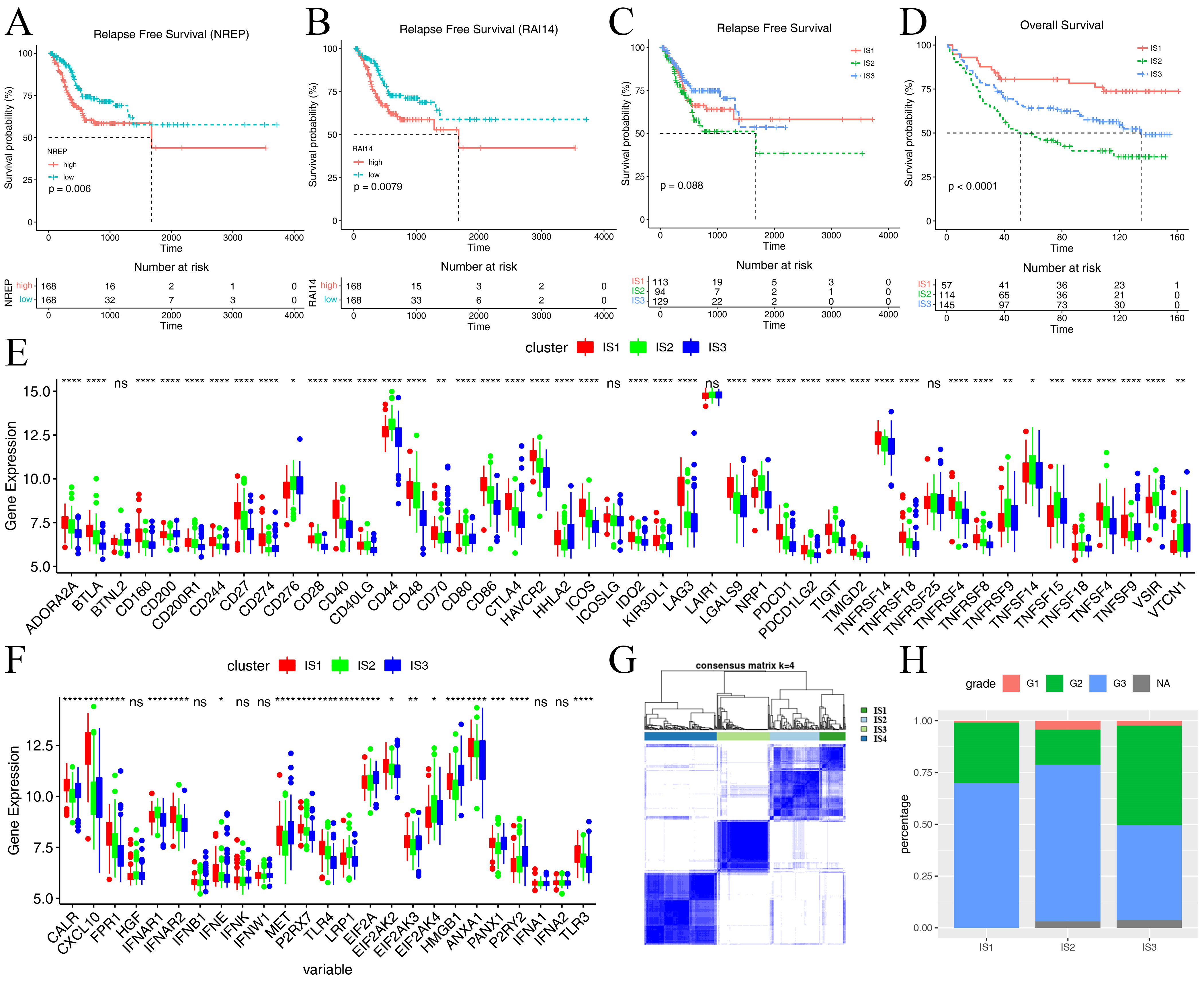

Supplement: Supplementary file 1 [file Image2.png]

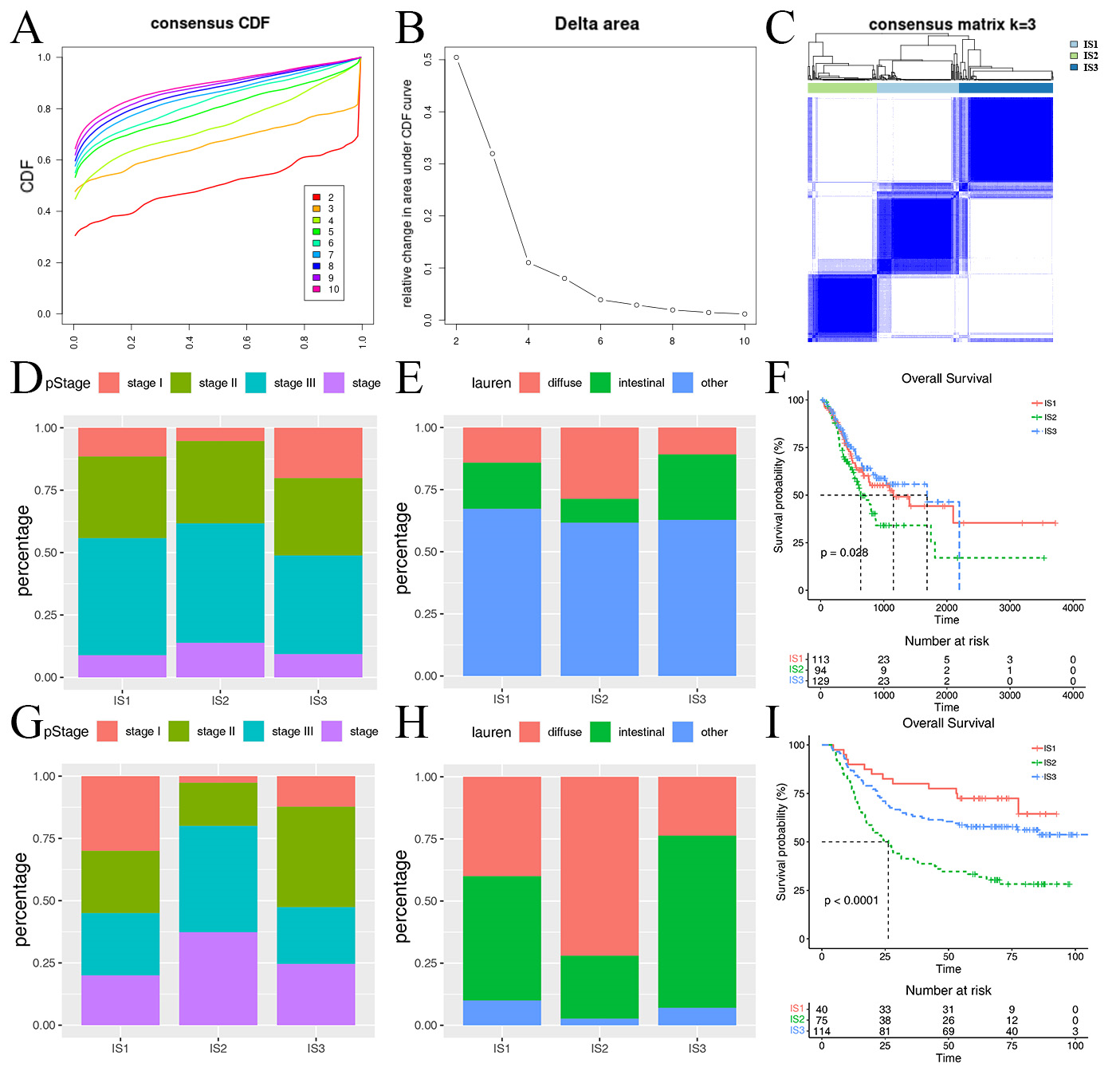

Supplement: Supplementary file 2 [file Image1.png]

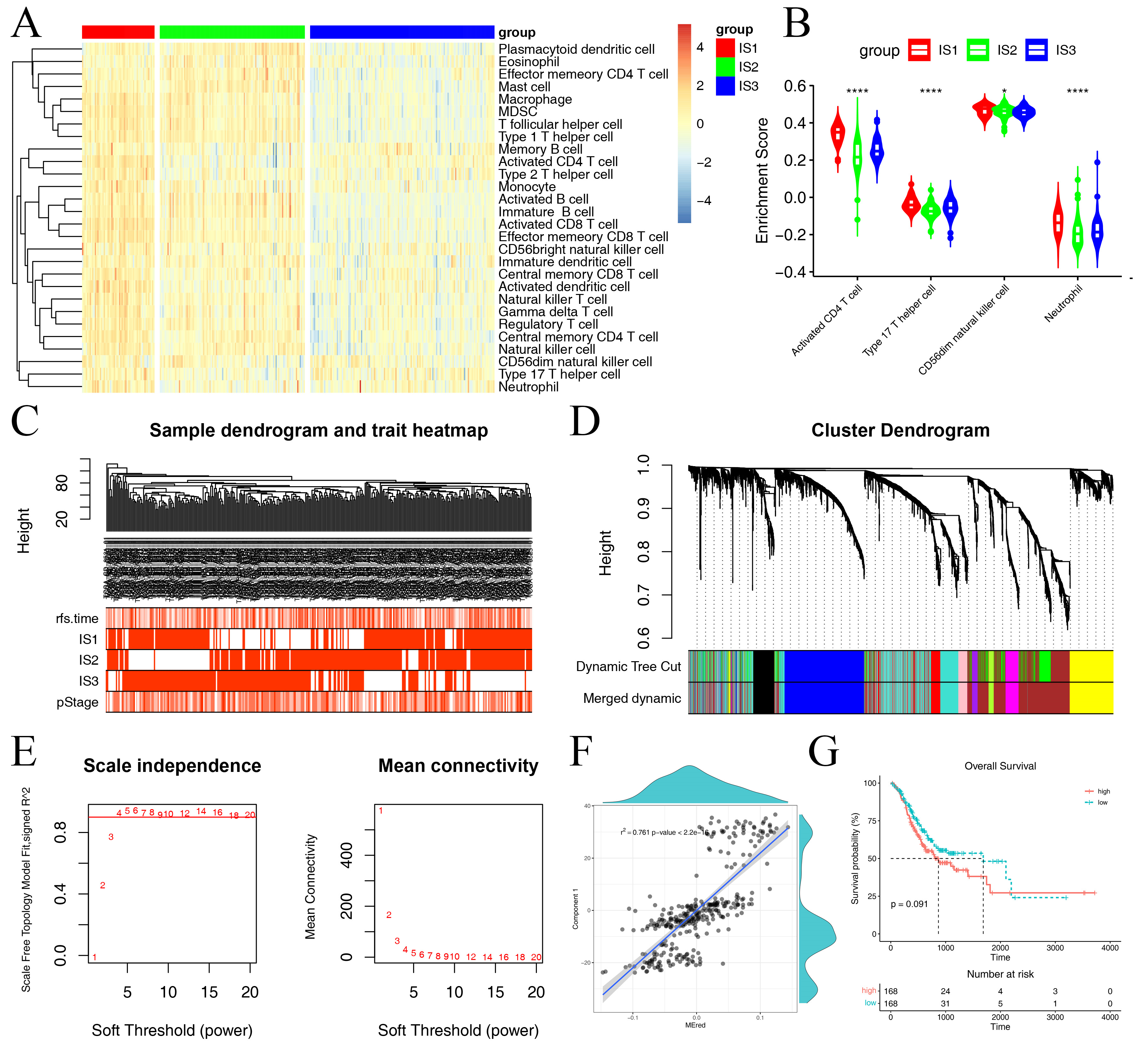

Supplement: Supplementary file 3 [file Image3.png]
